# Management of Type III Occipital Condyle Fractures

**DOI:** 10.3390/jcm13247639

**Published:** 2024-12-15

**Authors:** Jae Hyun Kwon, Anoop Sai Chinthala, Jonathan C. Arnold, Andrew J. Witten, Bradley N. Bohnstedt

**Affiliations:** Department of Neurological Surgery, Indiana University School of Medicine, 355 W. 15th St., Suite 5100, Indianapolis, IN 46202, USA; kwonjaeh@iu.edu (J.H.K.); aschinth@iu.edu (A.S.C.); jonarno@iu.edu (J.C.A.); andywitt@iu.edu (A.J.W.)

**Keywords:** occipital condyle fracture, cervical spine trauma, occipito-cervical fusion, orthosis

## Abstract

**Background/Objectives:** Occipital condyle fractures (OCFs) can be seen in around 4–19% of patients who suffer from cervical spine trauma. Anderson and Montesano system type III OCFs, which are avulsion fractures, are potentially unstable and operative. This study evaluates the management of type III OCFs at our institution over a 22-year period. **Methods:** This retrospective study reviewed all cases of type III OCFs at our institution from July 2001 to March 2023, identified via imaging reports. Using the in-house radiology imaging informatics system “Doris” (Dig Our Radiology Information System), reports containing the terms subluxation, avulsion, unstable, or type 3/III with occipital condyle, occipital condylar, occipital fx, or occipital fracture were collected. We also searched for Montesano type III/3 fracture. Electronic medical records were used to collect clinical and demographic data. Patients evaluated by the neurosurgical team with at least 1 month of follow-up were included in the analysis. **Results:** A total of 563 patients were identified with type III OCFs. A total of 56 patients met the inclusion criteria. The majority (91%, 51/56) were treated conservatively with cervical orthosis. A small subset (8.9%, 5/56) underwent occipito-cervical fusion. Three had concomitant unstable C1 fractures, while the other two had significant coronal deformity associated with their type III OCF. **Conclusions:** At our institution, type III OCFs are predominantly managed with cervical orthosis. Only those with an associated malalignment of the occipito-cervical joint underwent fusion. These findings suggest that most type III OCFs can be treated conservatively with orthosis once stability is confirmed with an upright radiograph.

## 1. Introduction

Occipital condyle fractures (OCFs) are observed in approximately 4–19% of patients who suffer from cervical spine trauma [1,2]. However, with the advancement of imaging modalities available and the increasing rate of high-energy traumas, the incidence of OCFs is now detected to be higher than that in the past [3,4]. Due to the occipital condyles’ anatomic importance to the cranio-cervical junction, an OCF could lead to a devastating neurological injury to the patient if the fracture is unstable. The diagnosis and classification of OCFs are typically conducted using computed tomography (CT) scans, while magnetic resonance imaging (MRI) can be used to assess ligaments that support the cranio-cervical junction such as the alar ligaments and tectorial membrane [4].

The Anderson and Montesano classification is the most commonly used method of categorizing OCFs: OCFs are categorized as type I, a comminuted fracture of the occipital condyle; type II, a skull base fracture extending to the condyle; or type III, an avulsion fracture of the condyle [5]. Type III OCFs have a higher likelihood of associated instability due to the involvement of the alar ligament [1,6]. These fractures have been reported to occur with an incidence 20–75% in patients with OCFs [4,7,8,9]. The concern with type III OCFs is that without surgical intervention, the fracture could remain unstable, potentially leading to complications such as chronic pain or neurological deficits [10]. Most recent studies advocate for conservative management, such as rigid cervical collars, in stable OCFs, but surgical treatment should remain a priority in unstable OCFs, such as type III OCFs [11,12]. There is controversy in the way we manage these fractures using the Anderson and Montesano classification, and it has been proposed that instead of this classification, only the presence of cranio-cervical misalignment should guide decision making [4,7,9].

At our institution, OCFs have been managed conservatively, including type III fractures. The purpose of our study was to investigate the need for advanced imaging techniques, such as MRI, or surgical fixation for these potentially unstable type III OCFs. We reviewed our institution’s management of type III OCFs over a 22-year period. We also analyzed the follow-up radiographic imaging of these patients to assess outcomes and treatment efficacy. Through this evaluation, we aim to contribute to the understanding and improvement of management strategies for type III OCFs.

## 2. Materials and Methods

Following institutional review board approval, this retrospective study enrolled adult patients (age ≥ 18) who had an Anderson and Montesano type III occipital condyle fracture identified on their imaging from July 2001 to March 2023. Radiology reports from this period were retrieved using the in-house radiology imaging informatics system, Dig Our Radiology Information System (DORIS), containing any combination of the following keywords: subluxation, avulsion, unstable, type 3/III occipital condyle, occipital condylar, occipital fx, or occipital fracture. Additionally, reports mentioning Montesano type III/3 were included. Duplicate records were manually identified and excluded. The inclusion criteria were patients having undergone at least one month of follow-up with the neurosurgery team and a confirmed type III OCF on imaging. Throughout the observational period, different providers followed varying follow-up protocols. However, all patients with documented follow-up had at least one month of follow-up. As a result, we established a minimum one-month follow-up as the inclusion criterion for our study. Our exclusion criteria were patients under the age of 18, patients without documented follow-up, and patients that did not have imaging on follow-up.

Electronic medical records were used to collect demographic data and clinical data, which included intracranial pathology, concomitant spine fracture, bracing type and duration, operative procedure if performed, MRI evidence of ligament tear, cranial nerve (CN) XI and XII pathology, presence of subsequent occipital neuralgia, suggestion of spinal cord injury, presence of vascular injury, and discharge disposition.

## 3. Results

We identified 563 patients, with 56 meeting our inclusion criteria and forming the cohort for this study. The mean age of the cohort was 44 years. A total of 33 patients (58.9%) were male, and 23 patients (41.1%) were female. This demographic was similar to the originally identified 562 patients, where the mean age was 46 years, and 63% and 37% of the patients were males and females, respectively. All the patients were placed in a rigid cervical collar (Table 1).

A total of 58.9% of the patients (33/56) had concomitant spine fractures, and all but one had concomitant cervical spine fractures. Due to this study’s retrospective nature, MRI was not obtained for all patients, but 46 out of the 56 patients had an MRI conducted, and 16 patients had an identifiable ligamentous injury. A total of 14 of these patients injured their alar ligament. A total of 21% of patients had suffered from some form or multiple forms of intracranial hemorrhage (Table 2): 12.5% had subdural hematoma (SDH), 16% had subarachnoid hemorrhage (SAH), 3.6% had intraparenchymal hemorrhage (IPH), and 5.4% had intraventricular hemorrhage (IVH).

A total of seven patients within the cohort underwent spinal fusion; however, one had C1-2 fusion for a C2 fracture, and the other had C2-T5 fusion for multiple cervical and thoracic fractures. Of the remaining five patients who underwent cranio-cervical fusion (OC fusion), three had a C1 concomitant fracture. Ultimately, two patients (3.6%) underwent OC fusion specifically for a type III OC fracture during the original hospitalization. No patients had OC fusion in a delayed fashion.

A total of 5.4% (3/56) of patients had signs of acute spinal cord injury, but based on a chart review, their injuries were due to concomitant fractures at other levels. None of the patients had cranial nerve XI, XII dysfunction, or signs of occipital neuralgia. A total of 16% (9/56) of the patients had blunt cerebrovascular injury, where all but two had injuries to the vertebral artery.

The mean duration of bracing for the cohort was 2.8 months. Upright plain films were obtained for all patients documenting stability prior to removing the rigid cervical collar. A total of 37.5% (21/56) patients were discharged to a rehabilitation facility, while the remaining patients were discharged home (Table 3).

## 4. Discussion

Our retrospective study assessed the institutional management of type III OCFs. The current literature on the management of OCFs is sparse, especially when looking specifically at type III fractures [1,3,4,6,7,8,9,10,11,12,13,14,15,16,17,18,19,20,21]. To the best of our knowledge, no study has looked specifically at the management of potentially unstable type III OCFs. Our in-house radiology imaging informatics system, DORIS, was able to identify 562 patients with type III OC fractures from 2001 to 2023, and ultimately, 56 patients met the inclusion criteria by having at least 1 month of follow-up with the neurosurgery team.

We report that 91% (51/56) of the patients were managed conservatively with a rigid cervical collar. This is consistent with the most recent data published on the management of all types of OCFs [11,12,13,14,15,17,18,19,20,21]. Even among the five patients who underwent OC fusion, indications for three of these patients were due to concomitant C1 fractures with associated C1 lateral mass displacement in relation to the C2 lateral mass. The calculated overhang was greater than 6.9 mm, classifying the fracture as unstable based on the Rule of Spence [22]. Additionally, MRI demonstrated transverse ligament disruption, concerning atlantoaxial instability. None of these patients had any permanent neurological issues localizing to the OCF based on a review of the electronic medical records. A total of 12.5% (7/56) of the patients had a blunt cerebrovascular injury to the vertebral artery, which is similar to what is reported in the literature [23]. All patients were cleared from their rigid cervical collar at 2.8 months on average. No patients had delayed OC fusion.

**Figure 1:**
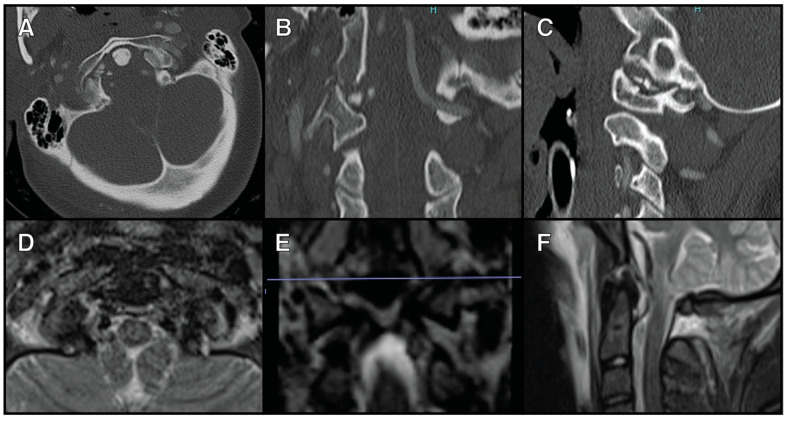
A 27-year-old female who presented after a motor vehicle collision with a right type III OCF and C1 lateral mass fracture (**A**) The axial view of a CTA scan showing the right type III OFC. (**B**) The coronal view of a CTA scan showing the right type III OFC. (**C**) The sagittal view of a CTA scan showing the right type II OFC. (**D**) The axial view of an MRI T2 STIR sequence showing right alar ligament disruption. (**E**) The coronal view of an MRI T2 STIR sequence showing right alar ligament disruption. (**F**) The sagittal view of an MRI T2 STIR sequence showing posterior ligamentous disruption.

Only 3.6% (2/56) of the patients were managed with OC fusion for their OCF alone. The first patient was a 27-year-old female who presented after a motor vehicle collision with a right type III OCF and C1 lateral mass fracture. There was a slight coronal deformity seen on CT scans, and the MRI of her cervical spine demonstrated the disruption of the right alar ligament, tectorial membrane, and posterior atlantoaxial ligament (Figure 1). This patient underwent an O-C3 fusion.

**Figure 2:**
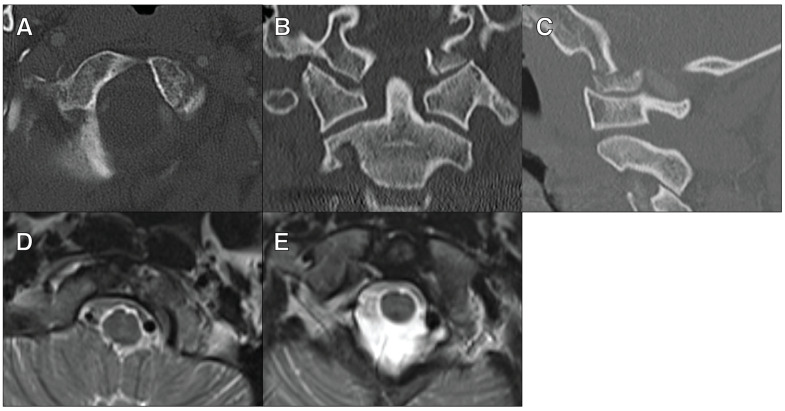
A 27-year-old male who presented after a fall from a scooter with a left type III OCF. (**A**) The axial view of a CT cervical scan showing the left type III OFC. (**B**) The coronal view of a CT cervical scan showing the left type III OFC and the coronal deformity. (**C**) The sagittal view of a CT cervical scan showing the left type III OFC. (**D**) The axial view of an MRI cervical T2 STIR sequence showing left alar ligament disruption. (**E**) The axial view of an MRI cervical T2 STIR sequence showing transverse cruciate ligament disruption.

The second patient was a 27-year-old male who presented after a fall from a scooter. He had a left type III OCF with obvious coronal deformity. MRI showed the disruption of the left alar ligament and the transverse cruciate ligament (Figure 2). He underwent an O-C2 fusion. Neither of these patients had any neurological deficits.

More recent research has pushed for the conservative management of all types of OCFs [12,21]. By assessing the radiographs for stability both at admission and at follow-up appointments, conservative management with a cervical collar is becoming the standard of care [12,13,14,17,21]. Our study’s approach is consistent with a conservative approach to those with type III OCFs. The vast majority of the patients in this study were treated conservatively for their OCF, and those that needed a surgical intervention had noticeable malalignment and various ligamentous injuries seen on imaging studies. Furthermore, 385 patients from the 562 did not meet the study criteria due to the lack of follow-up. Although detailed information regarding the functional/radiographic outcomes of these patients is unknown, it is most likely that the patients were asymptomatic since they did not pursue any further neurosurgical care.

The alar ligament and tectorial membrane contribute to cranio-cervical stability. Additionally, the atlanto-occipital joint capsules also have a significant role in cranio-cervical stability. Based on cadaveric study, the disruption of all elements is needed for cranio-cervical instability [24]. Type III OCFs are associated with the disruption of these structures and are thought to have higher potential for instability [4]. Despite this, our results support that the presence of ligamentous injury alone did not lead to surgical management in our cohort. A total of 34.8% of patients had ligamentous injury at presentation, but only two patients received OC fusion for a type III OCF. This highlights that the alignment of the cranio-cervical junction at the time of presentation has a greater yield in assessing the instability of type III OCFs.

Our study once again highlights the importance of individualized management based on radiographic and clinical findings to assess stability rather than relying on different methods of fracture classification. Past studies have suggested a similar approach to all types of occipital condyle fractures and that various OCF classification systems do not provide significant value to everyday clinical practice [4,7,9]. Our data suggest that type III OCFs can be managed conservatively with a rigid collar and plain radiographs upon follow-up. While MRIs are often required to rule out significant ligamentous injuries based on the Anderson and Montesano classification, our study indicates that this is not necessary for every patient. This approach has the potential to reduce healthcare resource utilization and alleviate the financial burden on patients. MRIs and surgical fixation should be reserved only for those with obvious malalignment or neurological deficits [12].

This study, however, does leave us with some limitations. The retrospective nature of this study prevented consistent data gathering. Despite a high number of patients with radiographic findings of type III OCFs, only 10% (56/562) of the patients met the study criteria due to the lack of adequate follow-up. This severely limited the size of the cohort. Due to the limited sample size in the surgical and conservative treatment groups, we were unable to perform a robust statistical comparison. The small sample size in the surgical cohort further limits the generalizability of our findings. There was also no protocol for the evaluation and outpatient management of type III OCFs. This led to an inconsistent initial imaging work-up, follow-up interval, and bracing duration. Addressing these issues may be beneficial for future studies with larger and more balanced sample sizes when investigating the length or even the need for a cervical collar for OCFs. Prospective, multicenter studies would better assess potential differences in outcomes between surgical and conservative treatment groups.

## 5. Conclusions

Anderson and Montesano type III occipital condyle fractures have been traditionally considered to be unstable. Advancements in imaging have provided better visualizations of the fractures and have influenced current treatment strategies. Subject to the limitation of being a single center’s experience, our study suggests that these fractures can be managed conservatively. MRI can be used for identification for ligamentous injury and a suggestion for surgical stabilization; however, it is not necessary for every patient. Malalignment with a type III OCF may also be an appropriate indication for surgery. Based on our results, we suggest the conservative management of type III OCFs with a cervical collar.

## Figures and Tables

**Table 1:** Patient demographics and baseline characteristic. Summary of demographic information and initial clinical characteristics of 56 patients with Anderson and Montesano type III occipital condyle fractures.

Characteristics	All Patients (n = 56)
Mean age, years ± STD ^1^	44
Gender, female, n (%)	23 (41.1)
Placement of rigid cervical collar, n (%)	56 (100)

^1^ STD = standard deviation.

**Table 2:** Clinical findings. Overview of clinical findings among patient cohort, including concomitant injuries, MRI results, and neurological assessments.

Clinical Findings	All Patients (n = 56)
Additional spine fractures, n (%)	33 (58.9)
—Only cervical spine fractures, n (%)	1 (3.0 ^†^)
MRI obtained, n (%)	46 (82.1)
—Ligamentous injury identified, n (%)	16 (34.8 ^†^)
—Alar ligament injury, n (%)	14 (30.4 ^†^)
Intracranial hemorrhage, n (%)	12 (21.4)
Signs of acute spinal cord injury, n (%)	3 (5.4)
—Associated with other level fractures, n (%)	3 (100 ^†^)
Cranial nerve XI, XII dysfunction, n (%)	0 (0)
Occipital neuralgia, n (%)	0 (0)
Blunt cerebrovascular injury, n (%)	9 (16.1)
—Vertebral artery injuries, n (%)	7 (77.8 ^†^)
—Other vascular injuries, n (%)	2 (22.2 ^†^)

^†^ Percent of parent category.

**Table 3:** Management and outcomes. Details of management approaches and outcomes, highlighting surgical interventions, bracing duration, and discharge destinations for studied patients.

Management/Outcomes	All Patients (n = 56)
Mean duration of bracing, months ± STD ^1^	2.8
Upright plain films before collar removal, n (%)	56 (100)
Surgical interventions	
—Total spinal fusions performed, n (%)	7 (12.5)
—C1-2 fusion for C2 fracture, n (%)	1 (14.3 ^†^)
—C2-T5 fusion for multiple fractures, n (%)	1 (14.3 ^†^)
—Cranio-cervical fusion (OC fusion), n (%) ^2^	5 (71.4 ^†^)
—For C1 concomitant fracture, n (%)	3 (60.0 *)
—For type III OC fracture, n (%)	2 (40.0 *)
—Delayed OC fusion, n (%)	0 (0 *)
Discharge destination	
—Rehabilitation facility, n (%)	21 (37.5)
—Home, n (%)	35 (62.5)

^†^ Percent of parent category total. * Percent of parent category total. ^1^ STD = standard deviation. ^2^ OC = occipital condyle.

## Data Availability

The data presented in this study are available on request from the corresponding author due to Health Insurance Portability and Accountability Act (HIPAA).
